# Ocean Acidification Alleviates Dwarf Eelgrass (*Zostera noltii*) Lipid Landscape Remodeling under Warming Stress

**DOI:** 10.3390/biology11050780

**Published:** 2022-05-20

**Authors:** Bernardo Duarte, Tiago Repolho, José Ricardo Paula, Isabel Caçador, Ana Rita Matos, Rui Rosa

**Affiliations:** 1 MARE—Marine and Environmental Sciences Centre & ARNET—Aquatic Research Infrastructure Network Associate Laboratory, Faculdade de Ciências da Universidade de Lisboa, Campo Grande, 1749-016 Lisbon, Portugal; micacador@fc.ul.pt; 2 Departamento de Biologia Vegetal, Faculdade de Ciências da Universidade de Lisboa, Campo Grande, 1749-016 Lisbon, Portugal; armatos@fc.ul.pt; 3 MARE—Marine and Environmental Sciences Centre & ARNET—Aquatic Research Infrastructure Network Associate Laboratory, Laboratório Marítimo da Guia, Faculdade de Ciências da Universidade de Lisboa, Avenida Nossa Senhora do Cabo 939, 2750-374 Cascais, Portugal; tfrepolho@fc.ul.pt (T.R.); jrpaula@fc.ul.pt (J.R.P.); rarosa@fc.ul.pt (R.R.); 4 Departamento de Biologia Animal, Faculdade de Ciências da Universidade de Lisboa, Campo Grande, 1749-016 Lisbon, Portugal; 5 BioISI—Biosystems and Integrative Sciences Institute, Plant Functional Genomics Group, Departamento de Biologia Vegetal, Faculdade de Ciências da Universidade de Lisboa, Campo Grande, 1749-016 Lisbon, Portugal

**Keywords:** seagrasses, lipidomics, fatty acids, ocean warming, ocean acidification

## Abstract

**Simple Summary:**

Expected climate change scenarios will have inevitable and important impacts on key foundation marine species such as seagrasses. This study was aimed to understand how the dwarf eelgrass *Zostera noltii* leaf lipid landscapes are altered under predicted ocean warming (+4 °C) and acidification (ΔpH 0.4) conditions. A severe reduction in the leaf total fatty acid (FA) content was observed in seagrasses individually exposed to hypercapnic or warming conditions, and this depletion was ameliorated under combined exposure to ocean warming and acidification conditions. The tested treatments also impacted the FA composition of all lipid classes, with warming exposure leading to decreases in polyunsaturated fatty acids (PUFAs). Galactolipid remodeling seems to have key roles in the physiological changes observed in seagrasses under these tested conditions, highlighting the higher impact of warming and that the proposed stress alleviation effect induced by increased water-dissolved CO_2_ availability. Neutral lipids were substantially increased under warming conditions, mainly with increases in C18 FA, impairing their use as substrates to maintain the osmotic balance of the cells. Nonetheless, the pace at which ocean warming is occurring can overcome the ameliorative capacity induced by higher CO_2_ availability, leaving seagrasses under severe heat stress beyond their lipid-remodeling capacity.

**Abstract:**

Coastal seagrass meadows provide a variety of essential ecological and economic services, including nursery grounds, sediment stabilization, nutrient cycling, coastal protection, and blue carbon sequestration. However, these ecosystems are highly threatened by ongoing climatic change. This study was aimed to understand how the dwarf eelgrass *Zostera noltii* leaf lipid landscapes are altered under predicted ocean warming (+4 °C) and hypercapnic (ΔpH 0.4) conditions. Warming and hypercapnic conditions were found to induce a severe reduction in the leaf total fatty acid, though the combined treatment substantially alleviated this depletion. The lipid discrimination revealed a significant increase in the relative monogalactosyldiacylglycerol (MGDG) content in both hypercapnic and warming conditions, allied to plastidial membrane stabilization mechanisms. Hypercapnia also promoted enhanced phosphatidylglycerol (PG) leaf contents, a mechanism often associated with thylakoid reinvigoration. In addition to changing the proportion of storage, galacto- and phospholipids, the tested treatments also impacted the FA composition of all lipid classes, with warming exposure leading to decreases in polyunsaturated fatty acids (PUFAs); however, the combination of both stress conditions alleviated this effect. The observed galactolipid and phospholipid PUFA decreases are compatible with a homeoviscous adaptation, allowing for the maintenance of membrane stability by counteracting excessive membrane fluidity. Neutral lipid contents were substantially increased under warming conditions, especially in C18 fatty acids (C18), impairing their use as substrates for fatty acylated derivatives essential for maintaining the osmotic balance of cells. An analysis of the phospholipid and galactolipid fatty acid profiles as a whole revealed a higher degree of discrimination, highlighting the higher impact of warming and the proposed stress alleviation effect induced by increased water-dissolved CO_2_ availability. Still, it is essential to remember that the pace at which the ocean is warming can overcome the ameliorative capacity induced by higher CO_2_ availability, leaving seagrasses under severe heat stress beyond their lipid remodeling capacity.

## 1. Introduction

Seagrass meadows are among the most productive and important habitats in marine coastal areas [1,2]. They are key primary producers that play an essential role in ecosystem functioning and architecture, and they are often referred to as ecosystem engineers. They sustain a key role in providing a variety of fundamental ecological and economic services, including nursery grounds, sediment stabilization, nutrient cycling, coastal protection, and blue carbon sequestration [3,4,5]. In the majority of coastal ecosystems, seagrass prairies are at risk due to increasing anthropogenic pressures, either in the form of direct pollution and impacts or due to human-activity-driven climate change [6]. Several studies have highlighted the importance of the conservation of these ecosystems (e.g., [7,8,9]), evidencing increasing seagrass habitat losses [9,10,11]. Several restoration efforts are currently being undertaken to recover the water quality of these key ecosystems through seagrass recovery and habitat protection [12,13] with a high degree of success [14,15]. Recent studies have shown that lessening anthropogenic stressors (nutrient loading, pollution, and habitat claiming) can also boost passive seagrass restoration, promoting increases in seagrass extent and density [16].

However, ongoing climate change has put these restoration efforts at risk. Climate change mitigation is now urgent [14] since the predicted increase in temperature [17] is expected to critically affect key physiological processes of seagrasses [18,19]. Atmospheric CO_2_ levels have been significantly increasing since the preindustrial period (280 ppm), reaching ~410 ppm at present, with worst-case scenario predictions indicating a further increase up to ~1100 ppm by 2100 [20]. Oceans are known to soak a large part of this atmospheric CO_2_, with the consequent unbalance of ocean carbonate chemistry and pH. Recent studies projected a decrease of the average ocean pH between 0.13 and 0.42 units by the end of the 21st century [20]. This increasing atmospheric CO_2_ also has the inevitable consequence of increasing global warming, and the temperature of the upper-oceans—the major sink of the excess global heat—has increased by between 0.09 and 0.13 °C over the last 40 years, with an expected increase rate of 0.2 °C per decade [20]. According to several studies, this increasing ocean warming is assumed to be the leading global threat to the health of seagrass ecosystems [9,21,22]. However, as previously observed for other plant species [23,24,25,26,27], this increasing CO_2_ fertilization can also alleviate plant stress. As CO_2_ is a limiting factor for marine plants, especially in warm environments, this ameliorating mechanism has also been observed for some seagrass species [28].

Beyond all the physiological adaptations that plants present to overcome abiotic stresses, fatty acid remodeling is also a common feature observed in several species [2,29,30]. In short, during membrane remodeling the lipid and fatty acid composition of membranes is altered by specific enzymatic pathways, which change the lipid and fatty acid composition of these compartments in order to cope with a certain stressor (biotic or abiotic) or environmental change [29,31]. These fatty acid remodeling events typically occur to stabilize chloroplastidial membranes and therefore maintain the good functioning of the Photosystem II (PS II) and include reductions in the concentrations of polyunsaturated fatty acids, such as alpha-linolenic acid (C18:3), and increased levels of trans-hexadecenoic acid (C16:1t) among others as adaptations to the new stressful environment [2,30]. Although while leaf fatty acid remodeling has been addressed in terrestrial halophytes under different stress conditions [1,29,30], the remodeling of the different lipid classes in seagrasses under predicted climate change scenarios still requires further investigation. Because seagrasses are important sources of essential fatty acids to marine-realm consumers, lipid remodeling is increasingly important, especially in the expected climate change scenarios.

The dwarf eelgrass *Z. noltii* has a wide random phase-exchange approximation distribution across the north-eastern Atlantic Ocean, from Norway to Mauritania and the Mediterranean coasts [32]. This ecosystem-engineer seagrass species forms intertidal meadows in transitional coastal areas, where the impact of climate change-related stressors occurs in combination with other anthropogenic-derived pressures [9], increasing its vulnerability [33,34,35]. Beyond its ecosystem-engineer role, *Z. noltii* represents an important direct and indirect feed source [36]. Its fatty acids represent one of the most important nutritional sources of energy, namely linoleic (C18:2, n-6) and α-linolenic (C18:3, n-3) acid, that are unique polyunsaturated fatty acids (PUFAs) that animals can only obtain through the diet [37]. These essential fatty acids are the precursors of long-chain PUFAs, and any disturbance at this level can affect the feeding ecology of the surrounding heterotrophs [38].

Within this context, the present work was aimed to evaluate the impacts of ocean warming and hypercapnic conditions in the seagrass leaf lipid and fatty acid composition and remodeling to disentangle the biochemical mechanisms underlying possible physiological changes. The potential of CO_2_ increase in the amelioration of warming-induced stress conditions was also studied, with a special emphasis on the seagrass lipidscape.

## 2. Materials and Methods

### 2.1. Experimental Conditions

*Zostera noltii* was collected (alongside with sediment) at a pristine area (38°29′18.42′′ N; 8°53′15.12′′ W, Caldeira de Tróia (Portugal) using a stainless-steel core (300 × 200 × 100 mm), as described in [39]. Subsequently, *Z. noltii* samples (*n* = 16) were transported to Laboratório Marítimo da Guia (Cascais, Portugal) and laboratory-acclimated for 30 days under the prevailing environmental conditions of the sampling site: salinity = 35 ± 1 (V2 refractometer, TMC Iberia, São Julião do Tojal, Portugal); seawater temperature = 18 ± 1 °C (TFX 430 Thermometer, WTW GmbH, Weilheim, Germany); and pH = 8.0 ± 0.1 (SG8—SevenGo proTM pH/Ion meter, Mettler-Toledo International Inc., Greifensee, Switzerland). After the initial acclimation period, seagrass was exposed to the following experimental conditions: (i) control (18 °C ad pH 8.0), (ii) acidification (18 °C and pH 7.6), (iii) warming (22 °C and pH 8.0), and (iv) combined warming and acidification (22 °C and pH 7.6), following IPCC’s RCP scenario 8.5. A total of 12 independent replicate tanks (i.e., 3 for each experimental condition) were randomly assigned. Experimental exposure was performed for 30 days. The monitoring and adjustment of pH values were automatically performed (Profilux 3.1, GHL, Kaiserslautern, Germany). The lowering of pH values was performed via the injection of certified CO_2_ (Air Liquide, Lisbon, Portugal) and upregulation through aeration using filtered atmospheric air (soda lime, Sigma-Aldrich, Saint Louis, MO, USA). Chilling systems (Frimar, Fernando Ribeiro Lda, Barcarena, Portugal) and submerged heaters (150 W, Eheim GmbH & Co. KG, Deizisau, Germany) were used to control seawater temperature during the entire experimental period. Further information related to additional abiotic culture conditions, monitoring, and assessment are described elsewhere [39], and a schematic can be found in Figure S1.

### 2.2. Thin Layer Chromatography (TLC) Lipid Separation and Fatty Acid Analysis

To achieve the necessary biomass for the lipid separation while ensuring all the fatty acids were detectable in all lipid fractions, three samples from each tank of each treatment were pooled into three combined replicates. Frozen seagrass leaf samples were boiled for 5 min to stop lipolytic activities [40]. Lipids were subsequently extracted using a mixture of chloroform/methanol/water (1:1:1) [41,42]. After vortexing, samples were centrifuged at 4000*× g* for 5 min and the chloroform phase was evaporated under nitrogen stream gas at 37 °C [29]. Lipids were resuspended in an ethanol/toluene (1:4) solution and stored at −20 °C under N_2_ atmosphere until analysis [29]. Lipid classes were separated by thin-layer chromatography (TLC), as described in [42] on silica gel plates (G-60, Merck), using a solvent mixture consisting of chloroform, methanol, acetone, acetic acid and water (100:20:40:20:8 *v*/*v*) for the separation of the different polar lipid classes from neutral lipids [29]. After migration, TLC plates were stained with primuline (0.01% *w*/*v* in 80% acetone *v*/*v*), and lipid bands were scraped off and methylated to fatty acid methyl esters (FAMEs) in a methanol/sulfuric acid (97.5:2.5) solution for 1 h at 70 °C. FAMEs were recovered by adding petroleum ether/ultrapure water (3:2), and the organic phase was collected after centrifugation before being dried and resuspended in hexane [43]. FAMEs were separated at 190 °C by gas chromatography via a 430 Gas Chromatograph (Varian) equipped with a hydrogen flame ionization detector, according to [44,45]. Heptadecanoic acid (C17:0) was used as an internal standard for quantification. Three replicates were analyzed per treatment. Several fatty acid ratios were calculated as follows:

$$PUFA/SFA=\frac{Polyunsatured\;Fatty\;Acids\;\left(\%\right)}{Saturated\;Fatty\;Acids\;\left(\%\right)}$$

$$UFA/SFA=\frac{Unsaturated\;Fatty\;Acids\;\left(\%\right)}{Saturated\;Fatty\;Acids\;\left(\%\right)}$$

$$Double\;Bond\;Index\;\left(DBI\right)=\frac{1\times monoenes+2\times dienes+3\times trienes}{100}$$

where PUFAs are all the fatty acids with 2 or more double-bonds, SFAs are all the fatty acids with one or more double-bonds, and UFAs are all the fatty acids with no double-bonds. Monoenes are considered to be fatty acids with only one double-bond, dienes are fatty acids with two double-bonds, and trienes are fatty acids with three double-bonds.

### 2.3. Statistical Analysis

Due to a lack of normality of homogeneity, univariate comparisons between treatments (lipid, fatty acid, and saturation ratio level) were performed using the non-parametric Kruskal–Wallis test and the ggpubr packages in R-studio (version 2021.09.0 Build 351). Since fatty acids are part of a metabolic chain, these were also analyzed using all fatty acids as a profile in a multivariate approach. Therefore, the fatty acid composition was analyzed using a multivariate statistical approach that has been proven to be efficient for the evaluation of the fatty acid profiles of different plant ecotypes [1,45,46,47]. A multivariate statistical analysis was conducted using Primer 6 software. Data from the relative total fatty acid composition in each treatment were used to construct a resemblance matrix based on Euclidean distances. The canonical analysis of principal coordinates (CAP) was used to generate a multivariate statistical model based on the relative fatty acid composition, using this profile as the modelling vectors for each lipid and treatment. CAP analysis also allows researchers to perform classification tests regarding the efficiency of FA-based models to classify and separate the different treatment groups. The permutational multivariate analysis of variance (PERMANOVA) was conducted to test the differences in lipid and fatty acid profiles between treatments [48].

## 3. Results

### 3.1. Total Leaf Fatty Acid

**Figure 1:**
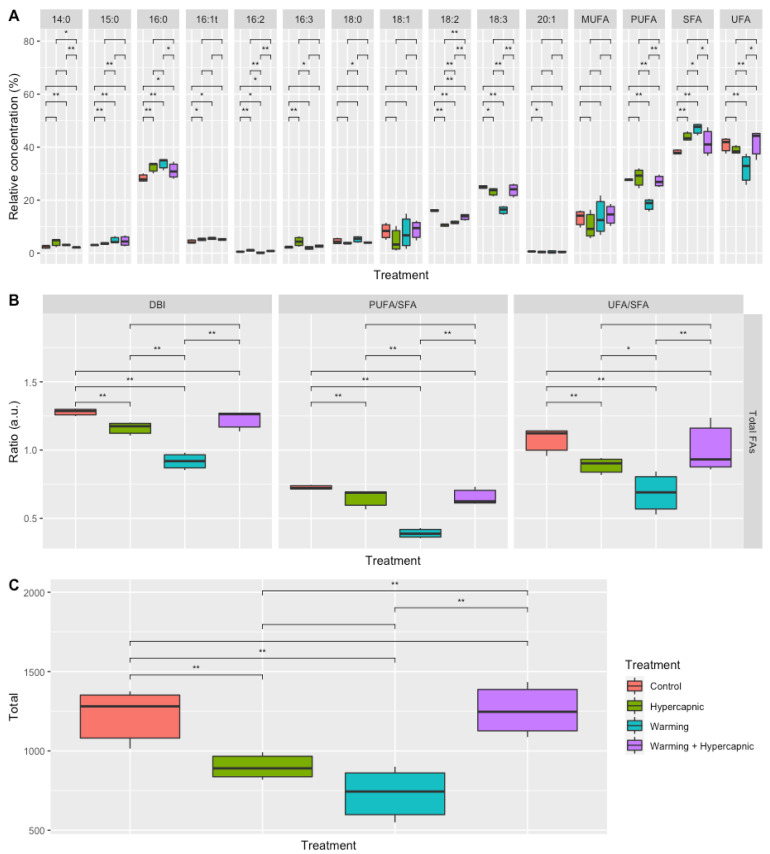
*Zostera noltii* leaf total fatty acid concentration (**A**), fatty acid indexes and ratios (**B**) (double-bond index, DBI; polyunsaturated to saturated fatty acid ratio, PUFA/SFA; unsaturated to saturated fatty acid ratio, UFA/SFA)), and total leaf fatty acid contents (**C**) under exposure to control, hypercapnic, warming and combined stress conditions (*n* = 3 per treatment; asterisks denote statistical differences among treatments at *p* < 0.05 (*) and *p* < 0.01 (**)).

Warming conditions produced the most significant impacts on the fatty acid profiles of the seagrass individuals (*p*_PERMANOVA t = 2.33_ = 0.037). It was also possible to observe significant increases in the relative 14:0, 15:0, 16:0, 16:1*t* and saturated fatty acid concentrations under warming-alone conditions (Figure 1A). The 16:2, 18:2 and 18:3 fatty acids, as well as the relative PUFA and UFA leaf concentrations, showed decreasing trends with the increasing temperature. Under hypercapnic conditions alone, significant increases in the relative 15:0, 16:0, 16:1, 16:2, 16:3, and SFA concentrations, as well as decreases in the relative leaf 18:2, 18:3 and 20:1 fatty acid concentrations, were observed. Under combined warming and hypercapnic conditions, significant increases in both 16:0 and 16:2 fatty acid leaf concentrations, as well as a decrease in the relative 18:2 abundance, were detected. These changes led to decreases in the total fatty acid DBI, PUFA/SFA and UFA/SFA ratios under exposure to warming and hypercapnic conditions (Figure 1B). Under the combined condition (warming and hypercapnia), no significant changes in the assessed fatty acid index and ratios were observed. Hypercapnic and warming conditions significantly reduced total leaf fatty acid concentration, while both factors combined resulted in values similar to those measured in the control plants (Figure 1B).

**Figure 2:**
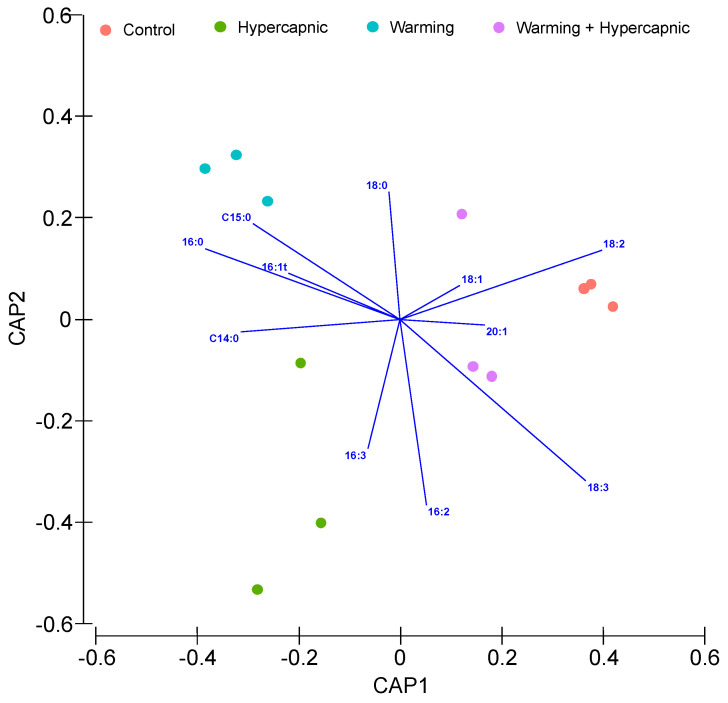
*Zostera noltii* leaf total fatty acid canonical analysis of principal (CAP) components of the samples exposed to control, hypercapnic, warming and combined stress conditions (*n* = 3 per treatment).

The leaf total fatty acid canonical analysis of principal (CAP) is shown in Figure 2. The cross-validation step presented a 100% classification efficiency of the samples belonging to the control and warming group, indicating a high specificity of the fatty acids as biomarkers of exposure to warming. This classification had a lower efficiency in samples exposed to hypercapnic conditions (66.7%). The leaf samples exposed to the combined stress conditions showed similar traits with the remaining samples.

### 3.2. Lipid Landscape Relative Composition

**Figure 3:**
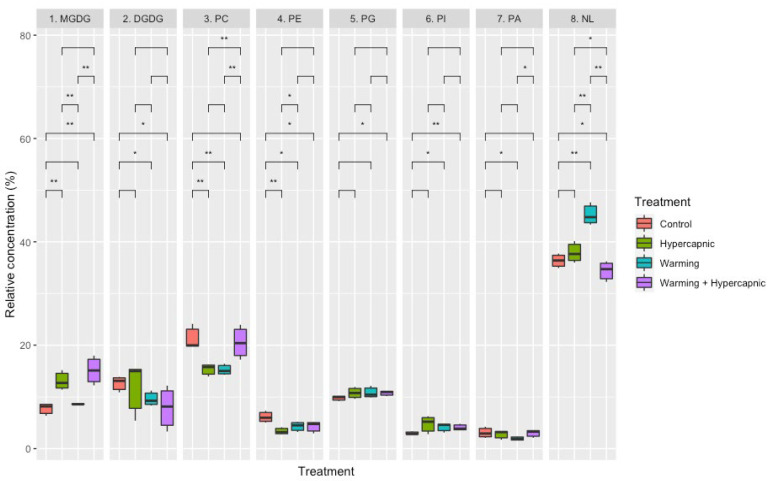
Relative *Zostera noltii* lipidscape composition (monogalactosyldiacylglycerol, MGDG; digalactosyldiacylglycerol, DGDG; phosphatidylcholine, PC; phosphatidylethanolamine, PE; phosphatidylglycerol, PG; phosphatidylinositol, PI; phosphatidic acid, PA; neutral lipids, NLs) under exposure to control, hypercapnic, warming and combined stress conditions (*n* = 3 per treatment; asterisks denote statistical differences among treatments at *p* < 0.05 (*) and *p* < 0.01 (**)).

**Figure 4:**
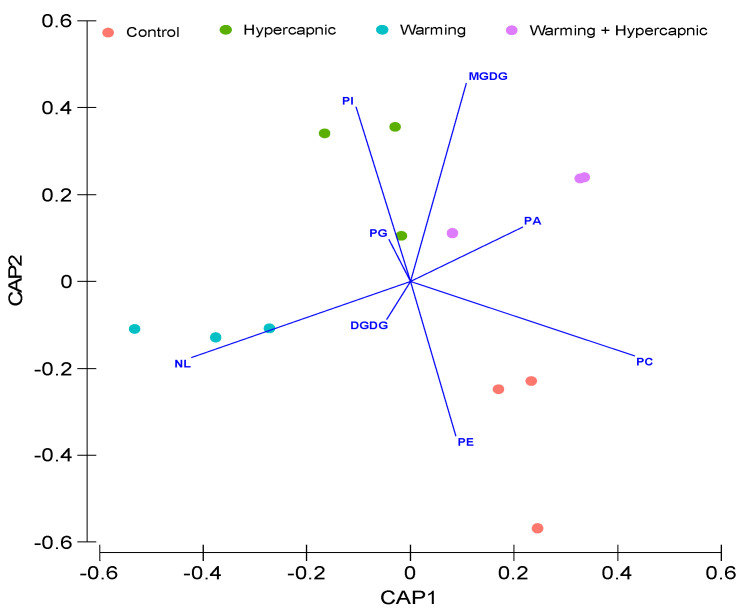
Relative *Zostera noltii* leaf lipid concentration canonical analysis of principal (CAP) components of the samples exposed to control, hypercapnic, warming and combined stress conditions (*n* = 3 per treatment; monogalactosyldiacylglycerol, MGDG; digalactosyldiacylglycerol, DGDG; phosphatidylcholine, PC; phosphatidylethanolamine, PE; phosphatidylglycerol, PG; phosphatidylinositol, PI; phosphatidic acid, PA; neutral lipids, NLs).

Warming alone produced the most significant changes in the lipid composition of *Z. noltii* leaves (*p*_PERMANOVA t = 3.89_ = 0.009), eliciting significant decreases in digalactosyldiacylglycerol (DGDG), phosphatidylcholine (PC), phosphatidylethanolamine (PE) and phosphatidic acid (PA) contents, as well as significant increases in phosphatidylinositol (PI) and neutral lipids (NL) contents. Hypercapnia alone led to an increase in the relative monogalactosyldiacylglycerol (MGDG) content and a decrease in PC concentration (Figure 3). Under the combined treatment, significant increases in MGDG, PG and PI contents were detected, while the relative DGDG, PE, and NL leaf concentrations showed significant reductions. A canonical analysis was also performed to evaluate the degree of specificity of these lipid signatures under the different treatments (Figure 4). Lipid class contents were able to discriminate the individuals exposed to control and warming conditions with 100% accuracy. A relatively lower efficiency (66.7%) was observed for samples exposed to the hypercapnia-alone and combined treatments. However, the relative leaf lipid concentration measurements had an overall 83.3% accuracy in depicting the treatment to which the samples were exposed.

### 3.3. Galactolipid Fatty Acid Profile

**Figure 5:**
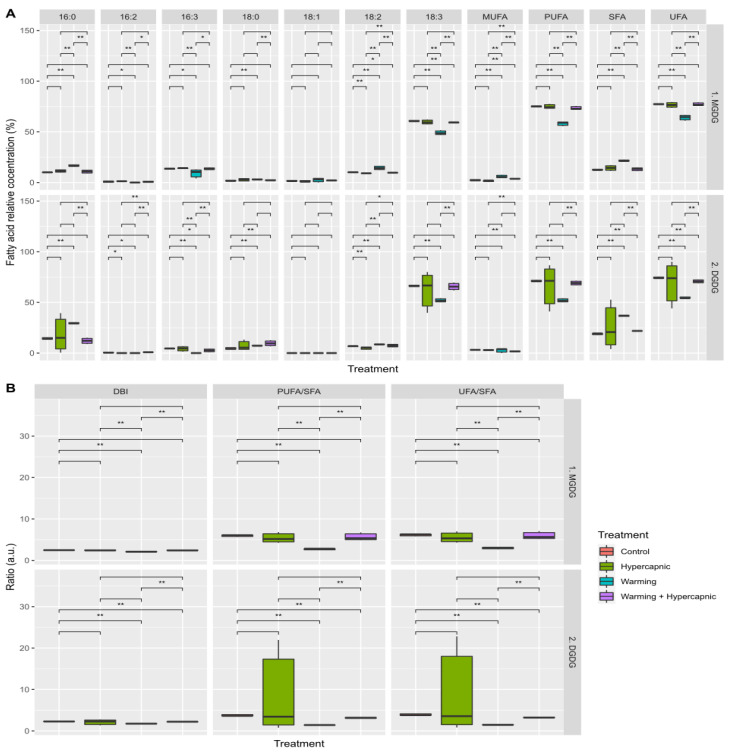
*Zostera noltii* leaf digalactosyldiacylglycerol (DGDG) and monogalactosyldiacylglycerol (MGDG) fatty acid composition and classes (monounsaturated fatty acids (MUFAs), polyunsaturated fatty acids (PUFAs), saturated fatty acids (SFAs), and unsaturated fatty acids (UFAs)) (**A**) and fatty acid indexes and ratios (double-bond index, DBI; polyunsaturated to saturated fatty acid ratio, PUFA/SFA; unsaturated to saturated fatty acid ratio, UFA/SFA) (**B**) under exposure to control, hypercapnic, warming and combined stress conditions (*n* = 3 per treatment; asterisks denote statistical differences among treatments at *p* < 0.05 (*) and *p* < 0.01 (**)).

Both the overall MGDG and DGDG fatty acid profiles were significantly affected by warming-alone conditions (*p*_PERMANOVA t = 10.28_ = 0.001), with significant increases in the leaf 16:0, 18:0, and 18:2 MGDG, as well as significant decreases in the 16:2, 16:3, and 18:3 MGDG. This resulted in decreases in the overall MGDG UFA and PUFA contents and increases in the relative MUFA and SFA contents (Figure 5A). Under hypercapnia-alone conditions, the relative 18:2 MGDG content showed a significant decrease (Figure 5A). Under the combined (warming and hypercapnia) treatment, MGDG showed a lower content in 18:2 and 18:3 fatty acids, leading to a significant increase in the MGDG MUFA concentration.

Under hypercapnia-alone conditions, the DGDG fatty acid profile showed significant decreases in its relative 16:2 and 18:2 concentrations. Warming-alone conditions induced increases in the 16:0, 18:0, and 18:2 DGDG fatty acids and the SFA abundance. Simultaneously, decreases in the relative 16:2, 16:3 and 18:3 fatty acid and DGDG PUFA and UFA contents were detected. Seagrass exposure to combined stress conditions revealed increases in the relative 18:0 and SFA abundances, while 16:3, MUFA and UFA showed significant decreases in relative concentrations (Figure 5A).

Hypercapnia-alone conditions did not significantly alter the MGDG and DGDG saturation ratios (Figure 5B). Warming-alone conditions significantly decreased all the assessed saturation traits in both MGDG and DGDG. Additionally, the combined treatment led to significant increases in all the DGDG saturation traits. Overall, exposure to combined hypercapnic and warming conditions induced leaf galactolipid profiles similar to those observed in the control individuals.

**Figure 6:**
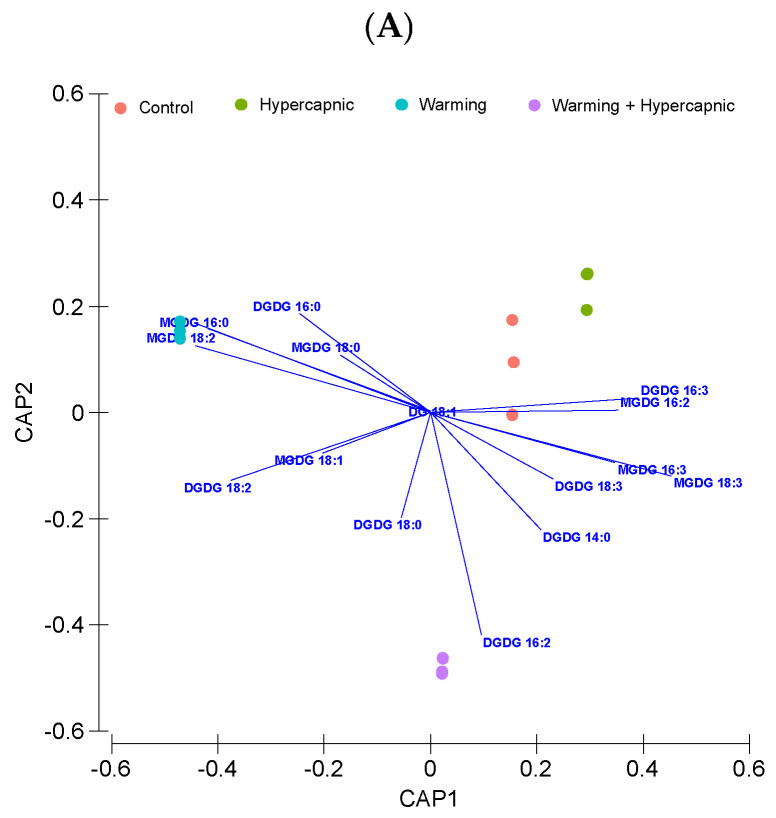

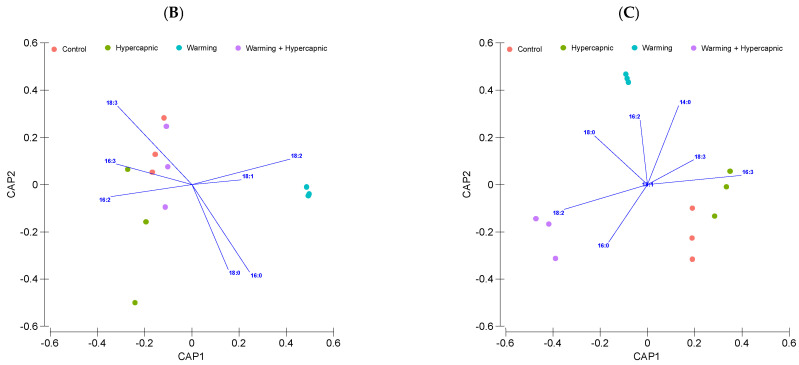
*Zostera noltii* leaf galactolipid (**A**), monogalactosyldiacylglycerol (MGDG) (**B**), and digalactosyldiacylglycerol (DGDG) (**C**) fatty acid relative concentration canonical analysis of principal (CAP) components of the samples exposed to control, hypercapnic, warming and combined stress conditions (*n* = 3 per treatment).

Observing the whole galactolipid fatty acid profile in a multivariate approach (Figure 6A) revealed an evident separation of the samples with a high degree of efficiency (75%), with only some misclassification between the samples exposed to control and hypercapnia-alone conditions. Taking a similar approach regarding the MGDG (Figure 6B) and DGDG (Figure 6C) fatty acid profiles individually, it was found that the overall classification efficiency showed some differences, with 66.7 and 83.3% for MGDG and DGDG, respectively. The reduced classification efficiency observed in the MGDG fatty acid profile was mostly due to misclassifications between control, hypercapnia-alone conditions, and combined treatments.

### 3.4. Phospholipid Fatty Acid Profile

**Figure 7:**
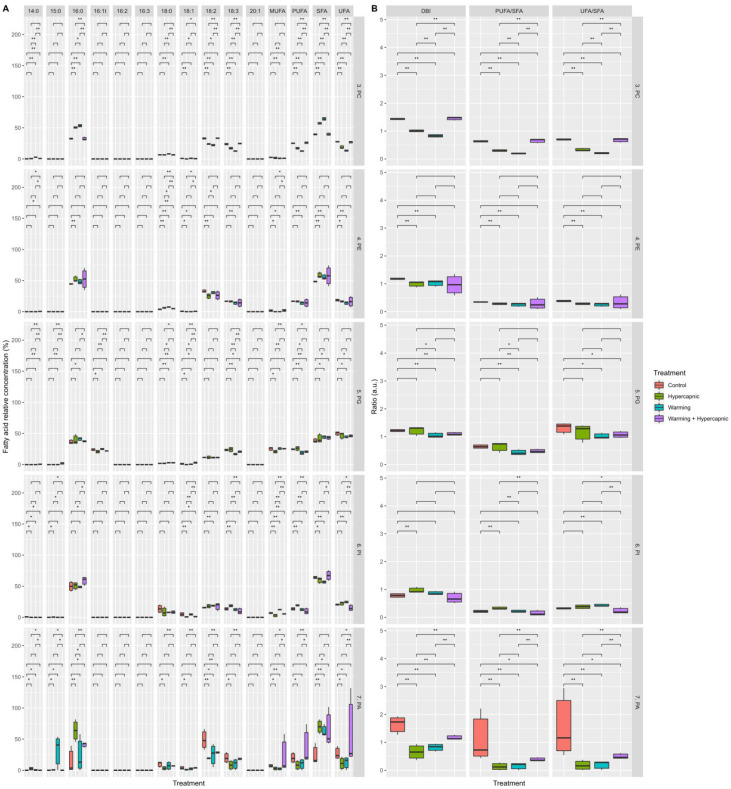
*Zostera noltii* phospholipid (phosphatidylcholine (PC), phosphatidylethanolamine (PE), phosphatidylglycerol (PG), phosphatidylinositol (PI), and phosphatidic acid (PA); numbers indicate the order of appearance of the lipid fractions in the TLC plate) fatty acid composition, saturation, and classes (monounsaturated fatty acids (MUFAs), polyunsaturated fatty acids (PUFAs), saturated fatty acids (SFAs), and unsaturated fatty acids (UFAs)) (**A**) and fatty acid indexes and ratios (double-bond index (DBI), polyunsaturated to saturated fatty acid ratio (PUFA/SFA), and unsaturated to saturated fatty acid ratio (UFA/SFA)) (**B**) under exposure to control, hypercapnic, warming and combined stress conditions (*n* = 3 per treatment; asterisks denote statistical differences among treatments at *p* < 0.05 (*) and *p* < 0.01 (**)).

A wide array of changes in the fatty acid composition of the different phospholipidic fractions were observed, so only the major differences in the most abundant fatty acid of each lipid fraction are focused on this section (Figure 7A). The phosphatidylcholine (PC) fatty acid profile under individually applied hypercapnic and warming conditions significantly differed from control conditions (*p*_PERMANOVA t = 9.46_ = 0.002 and *p*_PERMANOVA t = 11.63_ = 0.001, respectively). This lipid fraction was mostly constituted by 16:0, 18:2, and 18:3 fatty acids, comprising approximately 87–93% of the relative PC composition. The exposure to individually applied hypercapnic and warming conditions led to significant increases in the relative PC 16:0 and PC SFA compositions and significant reductions in the 18:2 and 18:3 fatty acid and PC UFA and PUFA contents (Figure 7B). No major differences were observed in the combined treatment.

The phosphatidylethanolamine (PE) fatty acid profile was only significantly affected under individually applied hypercapnic conditions (*p*_PERMANOVA t = 2.56_ = 0.05), with significant increases in the relative 16:0 and 18:2 contents. Individually applied warming conditions induced a significant decrease in the relative 18:3 content in PE. These fatty acids comprised 89–97% of PE acyl chains. PE MUFAs and UFAs showed significant decreases when hypercapnic and warming conditions were applied individually, while the inverse pattern was observed for the relative PE PUFA content. Hypercapnic and warming conditions (individually applied) induced reductions in all the PE indexes and ratios (Figure 7B). Warming conditions alone led to a significant change in the overall phosphatidylglycerol (PG) fatty acid profile (*p*_PERMANOVA t = 2.26_ = 0.05). The 16:0 and 18:3 fatty acids were significantly increased and reduced, respectively, in the plants exposed to warming conditions alone. This led to decreases in the PG PUFAs and PG UFAs and an increase in the relative PG SFA content under the warming-alone scenario. Trans-hexadecenoic acid (16:1t) and PG MUFAs showed significant decreases under hypercapnia-alone conditions. Regarding the saturation indexes and ratios (Figure 7B), the plants exposed to the warming and combined treatment showed significant reductions in all evaluated traits.

Analyzing the phosphatidylinositol (PI) fatty acid profile, the dominance of the 16:0, 18:2 and 18:3 fatty acids was again evident (Figure 7A). Hypercapnic conditions alone induced an increase in the relative 18:3 fatty acid concentration and significant reductions in PI MUFAs and SFAs. Warming conditions alone led to increases in the 18:2 fatty acid and relative PI MUFA and UFA concentrations, while the relative PI SFA concentration was significantly reduced. Regarding the saturation indexes, hypercapnic exposure (non-combined with warming) led to increases in the DBI and PUFA/SFA ratios, while under warming-alone conditions, the individuals revealed a higher PI UFA/SFA ratio (Figure 7B).

Only hypercapnic conditions significantly changed the overall phosphatidic acid (PA) fatty acid profile (*p*_PERMANOVA t = 2.87_ = 0.03), with significant increases in the relative PA 15:0 and 16:0 fatty acid contents accompanied by reductions in the relative 18:2 and 18:3 abundances; reductions in PA MUFAs, PA PUFAs, and PA UFAs; and inevitable increases in the SFAs (Figure 7A). These changes significantly reduced the DBI, PUFA/SFA and UFA/SFA ratios (Figure 7B). The individuals subjected to warming alone exhibited increases in the PA 15:0 fatty acid concentration and PA SFAs and UFAs, alongside a significant reduction in the relative 18:3 abundance, contributing to the reduction observed in all saturation indexes (Figure 7B). It is also worth noticing that the relative PA 16:0 and SFA abundances showed significant increases under the combined treatment and reductions in the 18:2 fatty acid PA saturation indexes (Figure 7B).

It is also important to reinforce that, in most of the cases and considering the relative fatty acid abundance, saturation classes, or ratios and indexes, the combined hypercapnic and warming treatment led to unsignificant changes compared to control conditions.

**Figure 8:**
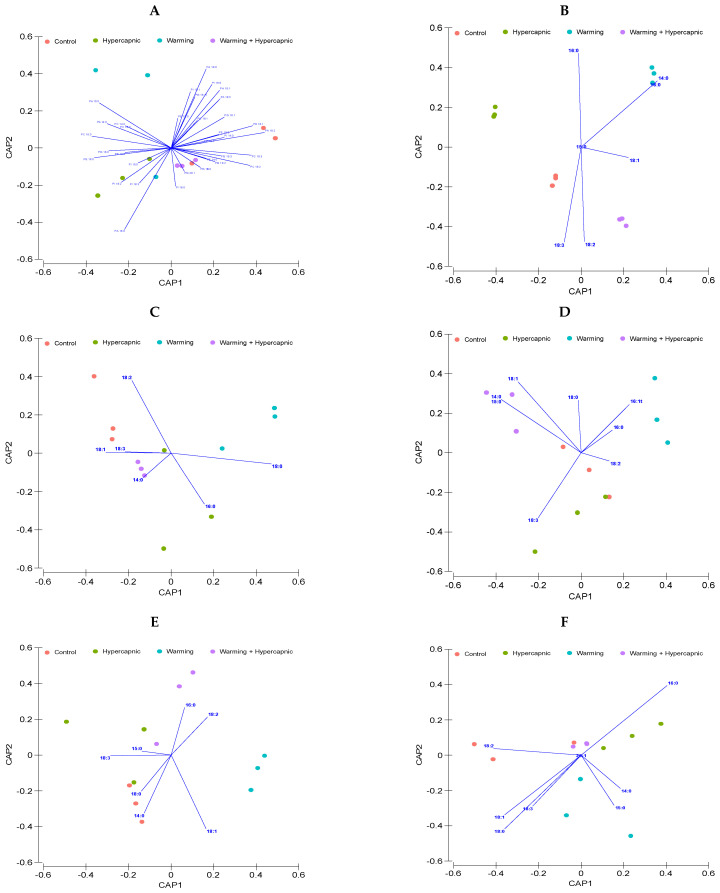
Relative *Zostera noltii* leaf phospholipid (**A**), phosphatidylcholine (PC) (**B**), phosphatidylethanolamine (PE) (**C**), phosphatidylglycerol (PG) (**D**), phosphatidylinositol (PI) (**E**), and phosphatidic acid (PA) (**F**) fatty acid concentration canonical analysis of principal (CAP) components of the samples exposed to control, hypercapnic, warming and combined stress conditions (*n* = 3 per treatment).

Within a multivariate approach (Figure 8A), the entire phospholipid fatty acid profile revealed some sample grouping with a high degree of efficiency (75%). Although some misclassification could be observed in the samples exposed to control, hypercapnic and warming conditions (individually applied), the relative phospholipid fatty acid concentration under the combined treatment showed a 100% classification efficiency. Considering the PC fatty acid profile (Figure 8B), the observed changes were highly specific to the treatments applied (canonical classification efficiency = 100%). PE and PI fatty acid profiles (Figure 8C,E) showed lower specificities towards the applied treatment (canonical classification efficiency = 66.7%), with only the PE fatty acid profiles of the individuals exposed to warming only conditions showing high degrees of specificity. Phosphatidylglycerol (PG; Figure 8D) fatty acid profile multivariate analysis revealed an overall 75% classification efficiency, with the PG profiles from the leaves of the seagrasses exposed to warming and the combined treatment showing high specificities towards the applied treatment (canonical classification efficiency = 100%). Similarly, PA fatty acid profiles also showed a 100% classification efficiency in the samples exposed to the combined treatment and an overall 75% classification efficiency when considering the PA profiles of all individuals (Figure 8F).

### 3.5. Neutral Lipid Fatty Acid Profile

**Figure 9:**
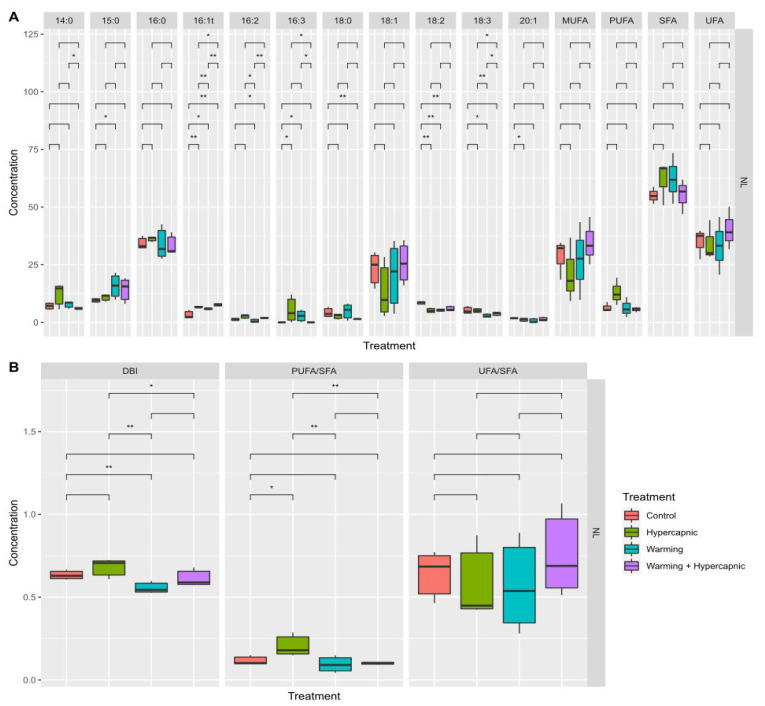
*Zostera noltii* neutral lipid (NL) fatty acid composition and saturation and classes (monounsaturated fatty acids (MUFAs), polyunsaturated fatty acids (PUFAs), saturated fatty acids (SFAs), and unsaturated fatty acids (UFAs)) (**A**) and fatty acid indexes and ratios (double-bond index, DBI; polyunsaturated to saturated fatty acid ratio, PUFA/SFA; unsaturated to saturated fatty acid ratio, UFA/SFA) (**B**) (*n* = 3 per treatment; asterisks denote statistical differences among treatments at *p* < 0.05 (*) and *p* < 0.01 (**)) of the samples exposed to control, hypercapnic, warming and combined stress conditions (*n* = 3 per treatment).

**Figure 10:**
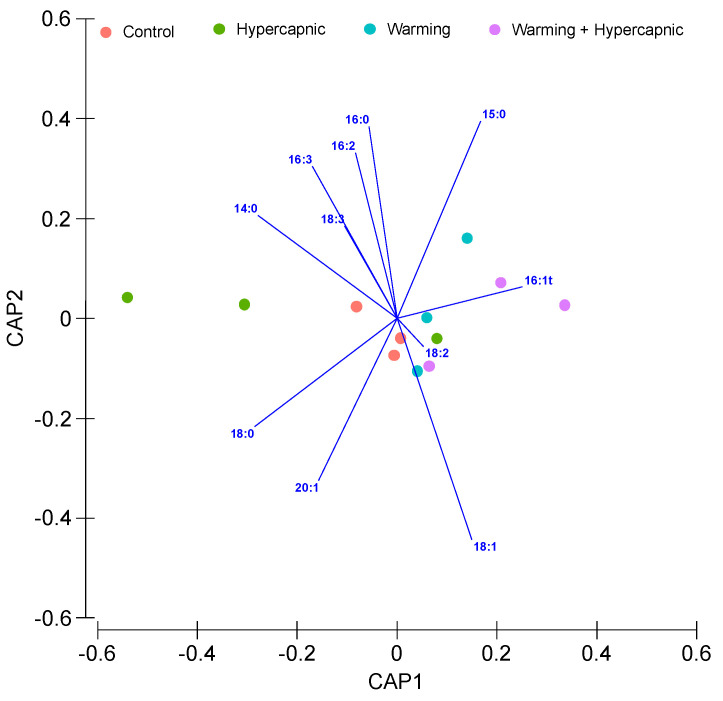
Relative *Zostera noltii* leaf neutral lipid fatty acid concentration canonical analysis of principal (CAP) components of the samples exposed to control, hypercapnic, warming and combined stress conditions (*n* = 3 per treatment).

The different experimental treatments did not elicit any significant changes in the overall neutral lipid fatty acid profiles compared to the control conditions (*p*_PERMANOVA_ > 0.05). Nonetheless, some individual differences could be observed in the relative fatty acid concentration (Figure 9A) and NL PUFA/SFA ratio (Figure 9B). Hypercapnia-alone conditions led to an increase in the relative 16:3 abundance and a significant reduction in 20:1 fatty acid. Regarding the warming effects when applied individually in the relative NL fatty acid concentration, significant increases in the 16:0, 16:1t, and 16:3 NLs were observed, and significant decreases in the relative 18:2 and 18:3 fatty acid abundances and NL DBI in this lipid fraction were also detected. Exposure to the combined treatment led to increases in the 16:1t and 16:2 NLs and reductions in the relative 18:0 and 18:2 concentrations. Considering the overall multivariate canonical analysis (Figure 10), a low classification efficiency was observed (58.3%), indicating an unspecific effect on the tested conditions.

## 4. Discussion

In the present work, three climate change scenarios were employed to evaluate how the leaf lipid remodeling of *Z. noltii* is affected by these factors, especially in terms of stress feedback and possible adaptation mechanisms. From the ecological point of view, the applied temperatures used to simulate a control scenario (18 °C) and a heat stress scenario (22 °C) were well within the range naturally experienced by this species—namely during marine heatwaves (MHWs), which are expected to become increasingly pervasive over the incoming years. Moreover, the experiment duration was also within the duration of MHWs observed in the Portuguese coast [49,50,51]. In this context, the experimental design was intended to mimic such events, which are known to have potentially devastating consequences for coastal ecosystems and for which there is no margin for adaptation. By specifically selecting the RCP scenario 8.5, it became possible to probe the current capacity of the species to cope with chronic exposure to the baseline conditions projected for a worst-case future and a relative present-day baseline at the collection site. In fact, selecting such a scenario was particularly pertinent considering the natural geographical range of the species, which is suggestive of a relatively robust coping capacity. Such resilience may, however, be mediated by phenomena of local adaptation [6]. With the underpinnings behind the thermal tolerance of this group still poorly understood, the present experimental design further provides valuable clues regarding the present-day biogeography of this species [6]. In this context, by selecting the most aggressive IPCC scenario and ecologically relevant baseline and exposure duration, this experimental design allowed for the collection information that is relevant for both present-day thermal anomalies (MHWs) and long-term climate change (including an increased prevalence of MHWs under milder scenarios/shorter timeframes). A similar rational can be applied to the tested CO_2_ conditions, which tend to considerably fluctuate in coastal areas (as such, RCP 8.5 projections are already within the range experienced by the species), with an exacerbation of such acidification events also expected to also take place over the incoming decades [52].

One of the first signs of stress detected in the seagrass leaf lipidscape was the severe reduction in the leaf total fatty acid content in the individuals exposed to hypercapnic and warming conditions individually, indicating the possible remobilization of energetically rich substrates (such as lipids and fatty acids) to counteract the imposed stress [29]. Another interesting aspect that was also possible to observe was the similar total fatty acid content of the individuals exposed to the combined (hypercapnia and warming) and control treatments. This may indicate that hypercapnia can alleviate negative warming impacts, in line with previous evidence that highlighted that increases in the medium CO_2,_ either atmospheric or seawater-dissolved, can constitute a favorable factor that enhances plant physiological fitness [24,25,26,27,53,54]. This fact has been attributed to increasing demand for NADPH following increasing CO_2_ levels [55] in order to enhance the electron transport rate under natural irradiance levels [56].

Going deeper into the lipid classes composition of the seagrass samples, the implications of these acclimation/response mechanisms can be disclosed. The increase in MGDG in both combined (hypercapnic and warming) and hypercapnic conditions suggested as a mechanism of membrane stabilization and increased interaction with possible heat-shock proteins (HSPs) [31]. The LHCII apoprotein of green plants, which contains two lipid molecules densely packed among other components, is assembled into trimeric complexes during the development of the thylakoid membrane [57,58]. This trimerization avoids proteolysis and enhances thermal stability [59]. The authors of [60] attributed the increased thermal stability of the light-harvesting complex II (LHCII) to MGDG and the interaction between its conic shape and the hourglass shape light-harvesting complex. This interaction allows MGDG to maintain a hexagonal lamellar phase, which induces a peripheral pressure, led by the galactose ring (DGDG presents a cylindrical shape caused by two galactose rings and forms a lamellar phase). Additionally relevant is the lipid environment involving plastoquinone A (Q_A_) and B (Q_B_), mainly constituted by MGDG. The increase in MGDG can contribute to the maintenance of the Q_A_ turnover, allowing for efficient electron transport throughout the thylakoid membrane [61]. The warming-alone-induced DGDG content reductions was in line with previous studies [31] and has been pointed out as a feedback mechanism to sustain the donor site of the PSII, usually sensitive to high temperatures [62]. In this case, this feedback mechanism is absent, and thus the PSII becomes more prone to warming, as it could be observed in terms of the photochemical performance of *Z. noltii* plants exposed to the same conditions [39]. PG increases under the combined conditions are often associated with thylakoid reinvigoration [63], thus once again supporting the thesis of stress-alleviation by increased CO_2_ availability.

Besides changing the proportion of storage, galacto- and phospholipids, the different treatments also impacted the FA compositions of all lipid classes. As a general trend, warming alone led to decreases in PUFAs, with the opposite trend being observed for saturated or, in some cases, MUFAs. Warming-induced decreases in PUFAs (and consequent increase in SFAs, as observed for these three lipid fractions) are compatible with a homeoviscous adaptation. Under this process, increases in SFAs have an objective to counteract the thylakoid membrane increasing fluidity (provoked by increasing temperature) and to maintain membrane stability under warming conditions [40,64]. As mentioned above, PG is the only phospholipid present in thylakoids, displaying a key role while enabling the correct organization of the light-harvesting complexes (LHC) antennae [65,66]. The increase in 18:0 was also compatible with a homeoviscous adaptation, which is aimed to maintain the integrity of the LHC [65,66] under warming-alone conditions. Once again, supplementation with CO_2_ (acidification alone or in combination with warming) apparently alleviated this stress, reducing the need for this homeoviscous adaptation. Regarding the synthesis of their membrane lipids, plastids are semiautonomous organelles, and this membrane adaptation could be intrinsic to the membrane or promoted by the importation of PUFA precursors from the endoplasmic reticulum [29]. It was suggested that a stress inhibition of lyso-phosphatidylcholine acyltransferase activity might result in the decreased import of lipids from the ER to the chloroplasts increasing extraplastidal 18:2 [67]. Under combined warming and acidification conditions, the fatty acid profile of a PC lipid fraction would exhibit a decrease in the relative 16:0 concentration alongside an increase in its relative 18:2 concentration [67]. However, this could not be observed in this case, revealing intrinsic MGDG, DGDG and PG remodeling. Another possible mechanism could be the synthesis of PE from PC. In this case, PE would show a similar profile and an increased DBI under stress conditions. This lipid is mainly found in the inner leaflet of the plasmaleme [68]. Phosphatidylethanolamine creates a more viscous membrane compared to PC [69]. Thus, an increase in DBI can be intrinsically connected to a need to increase membrane fluidity in a more efficient way to overcome warming stress.

Additionally, and according to previous studies [70], the decreased 18:3/18:2 ratio observed in the PE under warming-alone conditions indicates PE maintenance in the plasma membrane rather than an export towards the chloroplast. Moreover, our findings showed a reduction in the PE DBI under warming and hypercapnic conditions (individually), reinforcing that the observed MGDG, DGDG, and PG remodeling once again has an intrinsic origin. The higher 18:2 in the PI of the plants under warming conditions suggests that increases in long-chain polyunsaturated fatty acids (fatty acids with 18 carbons) may be essential for the vesicle bulk-flow endocytosis of the membrane area during plasmolytic membrane rearrangement under stress, indicating the existence of an adaptation mechanism based in PI fatty acid remodeling to overcome stress [71]. Once again, this mechanism seems to only be required under warming conditions alone, as it is absent in increased dissolved CO_2_ conditions. Warming-induced PA content may account for the membrane thermo-instability under heat stress [72]; it is a fast response mechanism because PA plays a role in plant stress signaling, with almost every environmental cue proved to trigger a rapid (seconds–minutes) PA response [73].

A warming-induced increase in NL indicates an increase in the cells lipid storage [29]. The predominant NLs in eukaryotic cells are triacylglycerols and stearyl esters, and they are a form of lipid storage mainly deposited into cytoplasmic lipid droplets (CLDs) [74]. The presence of lower relative concentrations of long-chain fatty acids (C18) in the NL compartment indicates a reduction in critical barrier functions required for osmotic maintenance [74]. These long-chain fatty acids (C18) can be used as substrates to fatty acylated derivatives of long-chain dialcohols that act to retain the water content at interfaces [74]. The lack of these functions may be based on the degree of stress observed in seagrass plants exposed to warming [39,75]. Again, increased CO_2_ availability (acidification conditions alone or in combination with warming) appears to ameliorate this storage need.

When analyzing the phospholipid and galactolipid fatty acid profiles, it became clear that the most pronounced effects under the different treatments were felt at the galactolipid level, as indicated by the high canonical efficiency. In physiological terms, this translates into a more relevant role of MGDG and DGDG remodeling underlying the physiological changes previously observed in the metabolism of the seagrasses under the tested conditions, especially in terms of energetic metabolism [39]. Additionally noteworthy was the enrichment in NL of plastidial lipids fatty acids (16:3 and 16:1t) in response to warming alone or in combination with acidification treatments. The accumulation of storage lipids and the plastidial acyl chain channeling to this lipid class is a transversal response of microalgae and higher plants [42,76] to stress conditions, although it has not yet been reported for seagrasses.

Moreover, when analyzing the differences between the warming and combined (warming and acidification) treatments, the galactolipids once again showed a higher degree of discrimination, highlighting not only the higher impact of warming at these lipids’ levels but also the proposed stress alleviation effect induced by increased CO_2_ availability. Furthermore, these differential impacts on the lipid landscape remodeling, specific to the lipid type and the applied treatment, also suggest that these lipid and fatty acid profiles are suitable biomarkers for these environmental stressors that may be used in the future to evaluate the impacts of climate change on seagrass ecosystems.

## 5. Conclusions

The present study clearly shows that warming elicits severe impacts on seagrass biochemical composition at the lipid and fatty acid levels, inducing substantial galactolipid remodeling. When subjected to warming alone, seagrasses present several signs of stress, namely the well-known mechanisms of homeoviscous adaptation and increased NL storage. However, ocean warming is expected to simultaneously occur with an increased ocean acidification scenario. From this work, it is possible to conclude that plants will benefit from higher CO_2_ levels to some extent, as this can enhance their photosynthetic metabolism. In fact, when *Z. noltii* plants were exposed to combined conditions of ocean warming and acidification, there was an amelioration of the stress signs with the remobilization of NL and a reduction in the need to adapt membrane fluidity. This may indicate that ocean acidification can reduce the degree of severity when these plants are subjected to ocean warming. Nonetheless, it is essential to remember that the pace at which the ocean is warming can overcome the ameliorative capacity induced by the higher CO_2_ availability, leaving seagrasses under severe heat stress beyond their lipid remodeling capacity.

## Data Availability

The data presented in this study are available on request from the corresponding author.

## Supplementary Materials

The following supporting information can be downloaded at: https://www.mdpi.com/article/10.3390/biology11050780/s1, Figure S1. Schematic representation of life support systems setup used in order to perform the experimental exposure of *Zostera noltii* to control, ocean warming and hypercapnic conditions. Temperature (18 °C and 22 °C); normocapnia (pH 8.0); hypercapnia (pH 7.6).
