# Supplementary material for: Ocean Acidification Alleviates Dwarf Eelgrass (Zostera noltii) Lipid Landscape Remodeling under Warming Stress
Source: Biology (Basel). 2022 May 20;11(5):780. doi: 10.3390/biology11050780 (PMC9138486; doi:10.3390/biology11050780)
Supplement: Supplementary file 1 [file biology-11-00780-s001.zip › biology-1687834-supplementary.pdf]

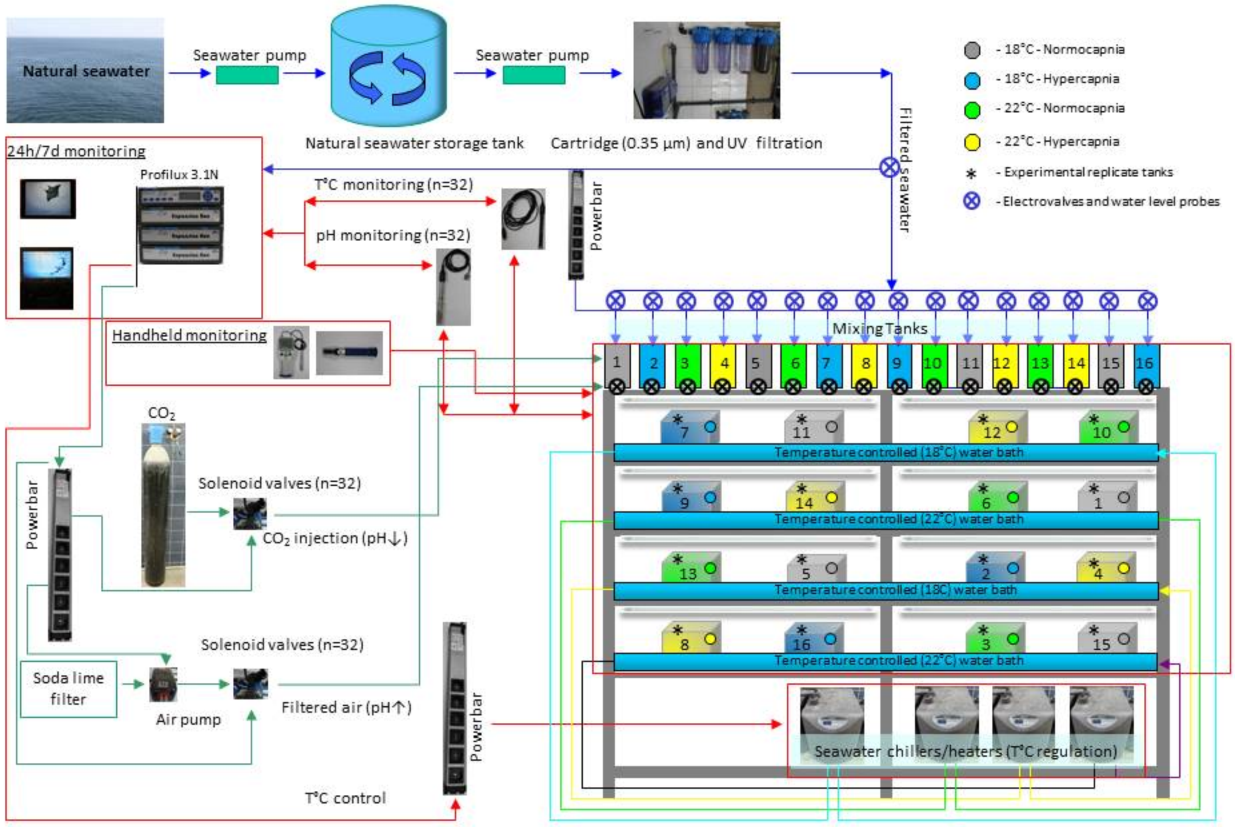

Supplementary Figure S1. Schematic representation of life support systems setup used in order to perform the experimental exposure of *Zostera noltii* to control, ocean warming and hypercapnic conditions. Temperature (18 °C and 22 °C); normocapnia (pH 8.0); hypercapnia (pH 7.6).

Supplementary Table S1. Seawater physicochemical parameters (temperature and pH) in all experimental setups. Salinity and temperature were measured daily and averaged per replicate aquarium over the whole experimental period. The combination of total alkalinity (A_T) and pH_T (pH total scale) was used to calculate carbonate system parameters [pCO_2 (carbon dioxide partial pressure), C_T (total inorganic carbon) and Ω Arg (aragonite saturation state)]. Values are represented as mean ± standard deviation.

Temperature	18 °C		22 °C	
pH	8.0	7.6	8.0	7.6
measured				
Temperature (°C)	18.0 ± 0.2	18.1 ± 0.2	21.9 ± 0.2	22.0 ± 0.2
Salinity	35.2 ± 0.9	34.5 ± 0.8	35.1 ± 1.1	35.4 ± 0.8
pH_T	8.00 ± 0.03	7.61 ± 0.03	8.01 ± 0.04	7.61 ± 0.02
A_T (µmol kg^{-1} SW)	1204.76 ± 105.04	1121.74 ± 104.55	1210.45 ± 105.61	1122.65 ± 99.03
calculated				
pCO_2 (ppm)	221.15 ± 3.74	580.42 ± 48.61	219.10 ± 4.90	595.01 ± 44.37
C_T (µmol kg^{-1} SW)	1051.64 ± 87.16	1071.18 ± 99.92	1036.53 ± 85.60	1062.25 ± 95.03
Ω Arg	1.29 ± 0.20	0.55 ± 0.08	1.50 ± 0.20	0.63 ± 0.07

Supplementary Table S2. Summary of two-way ANOVA performed in order to assess the effects of temperature (T) and pH over *Zostera noltii* endpoints (i-vi), following a 30-day experimental exposure to conditions simulating present day and future climate change scenarios. i) shoot density; ii) photophysiological parameters [electron transport rate (ETR); maximum PSII quantum yield (F_V/F_m)]; iii) pigments; iv) total chlorophyll and total carotenoid; v) carotenoid/chlorophyll ratio and vi) De-epoxidation state (DES). Significant statistical values are marked in bold (see main manuscript for more details on α).

	df	MS	F	p
**Shoot density** (α = 0.05)				
T	1	4556.25	63.83	**< 0.001**
pH	1	0.25	$3.50x10^{3}$	0.954
T x pH	1	121.00	1.69	0.217
Error	12	71.37		
**Photophysiological parameters** (α = 0.013)				
**ETR**				
T	1	2084.20	16.83	**< 0.001**
pH	1	412.60	3.33	0.070
T x pH	1	957.60	7.73	**0.006**
Error	182	123.90		
**F_V/F_m**				
T	1	0.57	41.59	**< 0.001**
pH	1	0.07	4.90	0.028
T x pH	1	$1.00x10^{-5}$	$< 1.00x10^{-5}$	0.984
Error	153	0.01		
**Pigments** (α = 0.013)				
**Chlorophyll *a***				
T	1	24621.00	6.66	**0.011**
pH	1	19286.00	5.22	0.024
T x pH	1	21951.00	5.94	0.016
Error	103	3694.00		
**Chlorophyll *b***				
T	1	1199.70	1.94	0.166

pH	1	3233.50	5.23	0.024
T x pH	1	555.90	0.90	0.344
Error	104	617.60		
**Total chlorophyll**				
T	1	35062.00	4.79	0.031
pH	1	32569.00	4.45	0.037
T x pH	1	34689.00	4.74	0.032
Error	103	7321.00		
**Pheophytin *a***				
T	1	9559.33	26.00	**< 0.001**
pH	1	284.52	0.77	0.381
T x pH	1	2054.85	5.59	0.020
Error	99	367.69		
**Pheophytin *b***				
T	1	2084.33	13.79	**< 0.001**
pH	1	68.47	0.45	0.502
T x pH	1	1008.07	6.67	**0.011**
Error	102	151.11		
**Auroxanthin**				
T	1	10809.20	16.88	**< 0.001**
pH	1	0.20	3.00×10^{-4}	0.986
T x pH	1	4384.10	6.85	**0.010**
Error	102	640.20		
**Antheraxanthin**				
T	1	1297.06	25.06	**< 0.001**
pH	1	501.82	9.70	**0.002**
T x pH	1	499.61	9.65	**0.002**
Error	103	51.75		
***β*-carotene**				
T	1	843.04	9.54	**0.002**
pH	1	564.80	6.39	**0.013**
T x pH	1	135.22	1.53	0.219
Error	105	88.39		
**Luteín**				
T	1	353.50	5.16	0.025
pH	1	323.31	4.72	0.032
T x pH	1	113.17	1.65	0.201
Error	104	68.43		
**Violaxanthin**				
T	1	25.34	1.15	0.286
pH	1	205.81	9.33	**0.003**
T x pH	1	19.60	0.89	0.348
Error	102	22.07		
**Zeaxanthin**				

T	1	363.70	3.71	0.057
pH	1	662.09	6.76	**0.011**
T x pH	1	109.10	1.11	0.294
Error	104	97.99		
**Total carotenoid**				
T	1	32097.00	13.45	**< 0.001**
pH	1	14599.00	6.12	0.015
T x pH	1	17497.00	7.33	**0.008**
Error	103	2386.00		
**Carotenoid/Chlorophyll ratio**				
T	1	1.50	9.20	**0.003**
pH	1	0.01	0.06	0.799
T x pH	1	1.39	8.53	**0.004**
Error	103	0.16		
**DES**				
T	1	0.046	7.03	**0.009**
pH	1	7.29×10^{-3}	1.11	0.294
T x pH	1	0.03	4.00	0.048
Error	104	6.55×10^{-3}		
